# Effect of Gibberellic Acid and Mechanical Scarification on the Germination and Seedling Stages of *Chenopodium quinoa* Willd. under Salt Stress

**DOI:** 10.3390/plants13101330

**Published:** 2024-05-12

**Authors:** Abderrahmane Nazih, Mourad Baghour, Abdesselam Maatougui, Kaoutar Aboukhalid, Basma Chiboub, Didier Bazile

**Affiliations:** 1 Multidisciplinary Faculty of Nador, Mohamed 1st University, B.P. 300, Selouane 60700, Morocco; mbaghour@hotmail.com (M.B.); basmachiboub@gmail.com (B.C.); 2 National Institute of Agronomic Research, CRRA Oujda, 10 Bd Mohamed VI, B.P. 428, Oujda 60000, Morocco; maatougui@hotmail.com (A.M.); k.aboukhalid@yahoo.com (K.A.); 3 CIRAD, UMR SENS, 34398 Montpellier, France; didier.bazile@cirad.fr; 4 SENS, CIRAD, IRD, Université de Paul Valéry Montpellier 3, 34090 Montpellier, France

**Keywords:** quinoa (*Chenopodium quinoa* Willd.), germination parameters, pre-treatments, salinity

## Abstract

Quinoa (*Chenopodium quinoa* Willd.) is a facultative halophyte renowned for its importance in enhancing food security, and it supports forage production across diverse climatic regions. The objective of this study is to examine the impacts of multiple pre-treatment methods on *C. quinoa* seed (Titicaca cultivar) germination parameters, identify the optimum pre-treatment to diminish the consequence of salinity, and promote the productivity of this crop, especially in marginal environments. For this purpose, a spectrum of sodium chloride (NaCl) concentrations spanning from 0 to 500 mM and gibberellic acid (GA3) concentrations ranging from 0 to 300 ppm were tested, and mechanical scarification (MS) was carried out. The effect of a combination of these pretreatment NaCl/GA3 and NaCl/MS on the germination parameters of *C. quinoa* seed was also investigated. The results showed that the total germination, vigor index, and germination index decreased progressively with an increase in salinity. Hence, salinity exhibited a notable influence on most germination parameters. Moreover, seeds scarified with 500 mM of NaCl negatively affected all measured parameters. In contrast, gibberellic acid applied at 200 ppm was effective on most of the parameters measured, particularly under 100 mM of NaCl. These findings indicate that immersing seeds in gibberellic acid could mitigate the adverse impacts of salinity.

## 1. Introduction

In many regions worldwide, salinity stands as one of the longstanding and consequential environmental challenges, significantly restricting crop production [1]. The expansion of populations and intensified agricultural outputs have led to a decline in freshwater resources available for irrigation, prompting the utilization of alternative water sources characterized by diminishing quality [1,2,3].

In Morocco, soil salinity presents a substantial challenge to agricultural endeavors, with an estimated extent of affected soil areas reaching 1.148 million hectares [4], and approximately 85% of the zones are categorized as arid [5]. Regarding Morocco, agriculture is reliant upon climatic parameters, which are characterized by irregular precipitation, although groundwater and surface resources are important for socio-economic improvement [6]. However, saline irrigation affects productivity, degrades soil structure, and reduces fertility [2].

The germination of seeds constitutes a crucial stage in the life cycle of plants, and it is susceptible to diverse abiotic factors, particularly widespread in arid and semi-arid regions, and encompass factors such as salinity and water scarcity, which can reduce the osmosis of soil water and water potential gradients and restrict seed water absorption [7,8]. This confirms a negative correlation with the germination rates of certain species [9,10] and limits seedling establishment.

Currently, an alternative approach involves the cultivation of halophytic crop species that are capable of withstanding elevated levels of soil salinity, exemplified by Quinoa (*Chenopodium quinoa* Willd.). Quinoa, classified as a pseudo-cereal, demonstrates minimal input requirements, is drought- and stress-tolerant [11], is adapted to adverse climates with high nutritional value, and is regarded as a viable alternative crop for ensuring food security [12,13,14,15].

Some pretreatments such as phytohormones, scarification, and others are methods that break seed dormancy and enhance seedling emergence and growth when all the necessary conditions are accomplished [16]. Moreover, scarification is among the techniques employed to disrupt the physical dormancy of seeds, allowing the seed to exchange water and gases with its environment and promoting embryo development [17].

For crops grown in saline conditions, the yield and growth of these crops can be improved by pre-treating seeds with phytohormones [18]. Ref. [19] propose employing various application methods, such as soaking seeds prior to sowing and spraying with a salicylic acid solution, in order to safeguard these crops against salt stress through the induction of a large range of processes implicated in the stress tolerance mechanism. The use of gibberellic acid is also useful in reducing salinity stress [20].

In Morocco, a great part of agricultural regions is influenced by different climatic constraints, such as irregular rainfall and soil salinization processes. Indeed, approximately 5% of land is currently afflicted by varying degrees of salinity, acting as a constraining factor that impacts agricultural productivity, particularly in arid and semi-arid regions [21]. Therefore, understanding the germination behavior of salt-tolerant plants with high economic values, such as *C. quinoa*, can guide successful cultivation practices of this crop.

The objective of this study was to evaluate the impact of salt stress and different pretreatments, including gibberellic acid (GA3), mechanical scarification (MS), and the combined treatment of NaCl/GA3 and NaCl/MS. The aim of this study is to assess the influence of various pre-treatments (GA3 and MS) on the germination parameters and initial growth of quinoa seedlings, with the goal of enhancing salt tolerance in quinoa seeds. Furthermore, to our knowledge, there are no similar studies investigating the effects of various pretreatments (GA3 and MS) and their interactions with salinity with respect to the seed germination of quinoa in Morocco.

## 2. Results

The results of the statistical analysis are summarized in Table 1, and all results are presented as mean values ± standard deviation. The figures illustrate these results graphically, while the tables provide a detailed view of the data. All values in these tables and figures are presented as the mean ± standard deviation.

### 2.1. Total Germination, Vigor Index, Mean Germination Time, and Germination Index

Table 1 shows no significant effect of the pretreatments on the total germination rate and mean germination rate of *C. quinoa* seeds. However, the gibberellic acid at 100 ppm had a highly significant effect on the vigor index (*p* ≤ 0.01).

Moreover, salinity had a very highly significant effect on the total germination rate, vigor index, and germination index of quinoa seeds (*p* ≤ 0.001).

**Figure 1:**
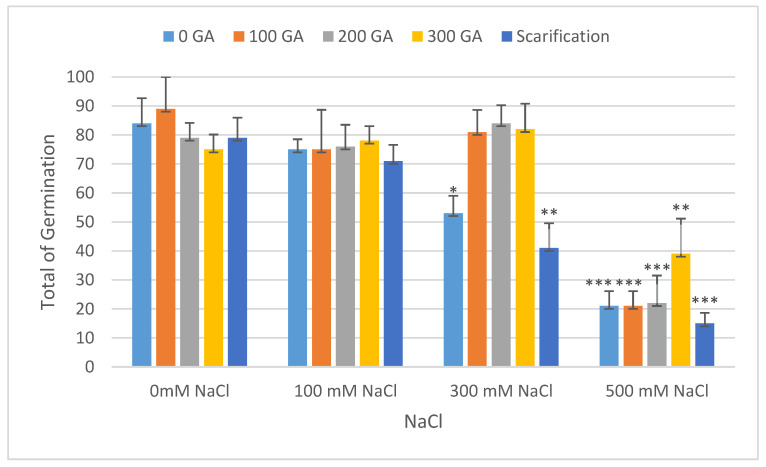
Effect of salinity, pre-treatment, and their interaction on total germination rate (TG). Error bars indicate standard deviation, and bars with *, **, and *** indicate statistically significant differences compared to the control when *p* ≤ 0.05, *p* ≤ 0.01, and *p* ≤ 0.001, respectively. Means without * are not statistically significant differences according to Tukey’s test.

**Figure 2:**
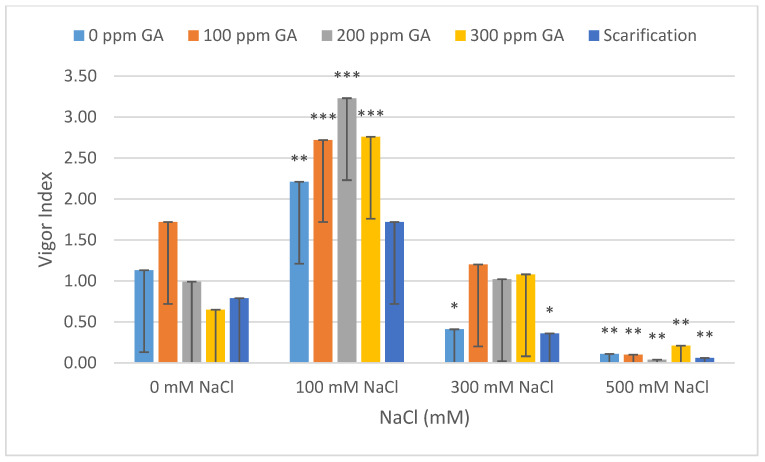
Effect of salinity, pre-treatment, and their interaction on the vigor index (VI). Error bars indicate standard deviation, and bars with *, **, and *** indicate statistically significant differences compared to the control when *p* ≤ 0.05, *p* ≤ 0.01, and *p* ≤ 0.001, respectively. Means without * are not statistically significant differences according to Tukey’s test.

**Figure 3:**
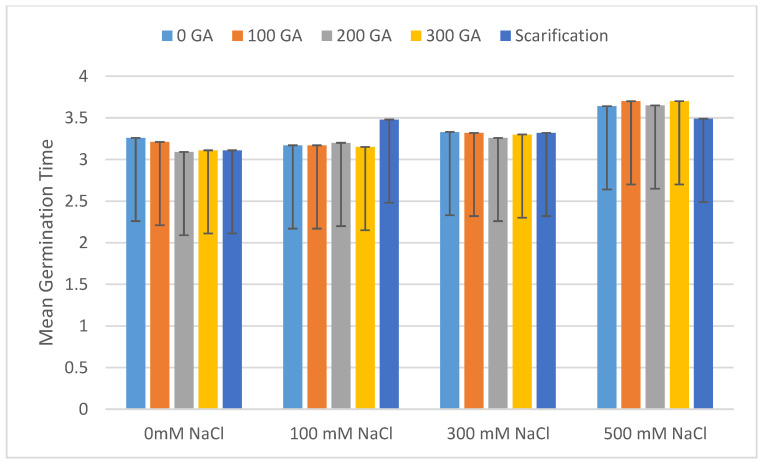
Effect of salinity, pre-treatment, and their interaction on mean germination time (MGT), error bars indicate standard deviation. Means without * are not statistically significant differences according to Tukey’s test.

**Figure 4:**
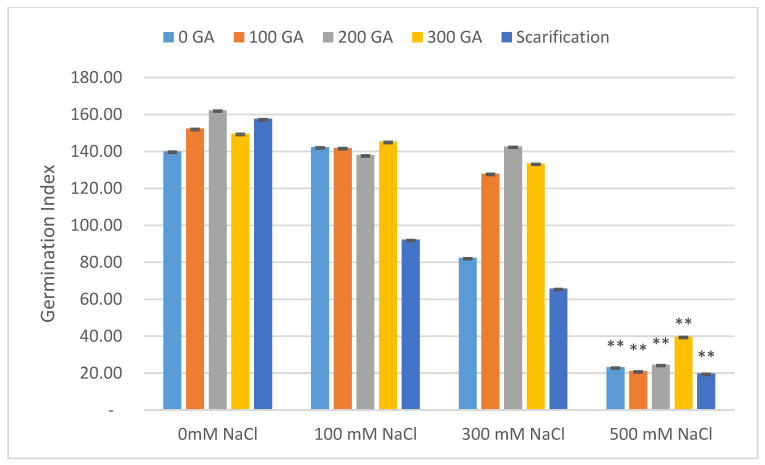
Effect of salinity, pre-treatment, and their interaction on germination index (GI). Error bars indicate standard deviation and bars with ** indicate statistically significant differences compared to the control when *p* ≤ 0.01. Means without * are not statistically significant differences according to Tukey’s test.

The total germination rate decreased with increasing NaCl concentrations, and the mean value of total germination decreased by 8.67%, 30.67%, and 62.67% under 100, 300, and 500 mM NaCl, respectively (Figure 1 and Table 2). In addition, at 100, 300, and 500 mM NaCl, the mean value for the vigor index had increased by 1.08 and decreased by 0.72 and 1.02, respectively (Figure 2 and Table 2). The mean germination time (day) increased by 0.06 and 0.38 and decreased by 0.09 at 300, 500, and 100 mM NaCl, respectively (Figure 3 and Table 3). The germination index increased by 1.32 and decreased by 58.64 and 117.87 under 100, 300, and 500 mM NaCl, respectively (Figure 4 and Table 3).

Furthermore, the data obtained showed that the interaction of pretreatments and salinity had no significant effect on the total germination, vigor index, mean germination time, and germination index.

According to Table 2 and Table 3, mechanical scarification with 500 mM NaCl showed a maximum decrease in the mean values for total germination, the vigor index, and the germination index (68.67%, 1.07, and 121.29, respectively). In addition, the maximum increase in total germination was observed at 100 ppm gibberellic acid without saline treatment (5.33%). For the vigor Index, the maximum increase (2.10) was observed at 200 ppm GA3 under 100 mM NaCl.

At 200 ppm GA3, in the absence of NaCl, there was a maximum decrease (0.15 day) in the mean values for mean germination time and a maximum increase (20.26) for the germination index (Table 3).

### 2.2. Fresh Weight, Dry Weight, Fresh Length, and Dry Length

According to Table 1, there was no significant effect of GA3 and scarification on the fresh weight, dry weight, fresh length, and dry length of quinoa seedlings after 5 days of germination. A similar result was observed between the interaction of pretreatments and salinity on fresh weight, dry weight, fresh length, and dry length. Concerning salinity, no statistical difference was observed with respect to dry weight; however, there was a significant effect on the fresh weight, fresh length, and dry length of quinoa seedlings.

**Figure 5:**
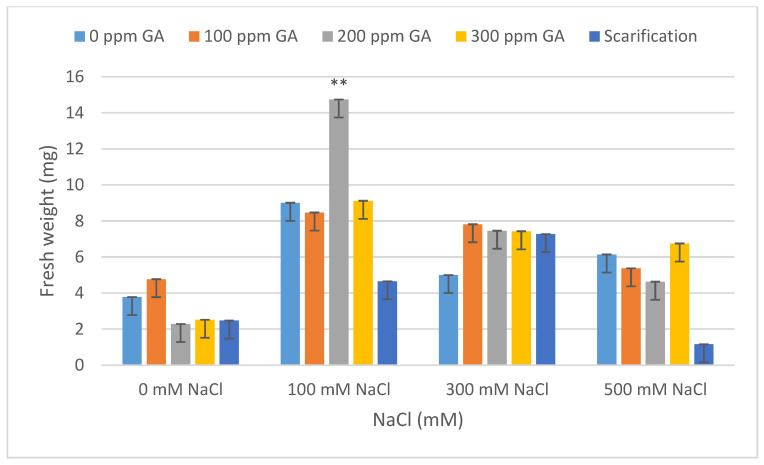
Effect of salinity, pre-treatment, and their interaction on fresh weight (FW). Error bars indicate standard deviation and bars with ** indicate statistically significant differences compared to the control when *p* ≤ 0.01. Means without * are not statistically significant differences according to Tukey’s test.

**Figure 6:**
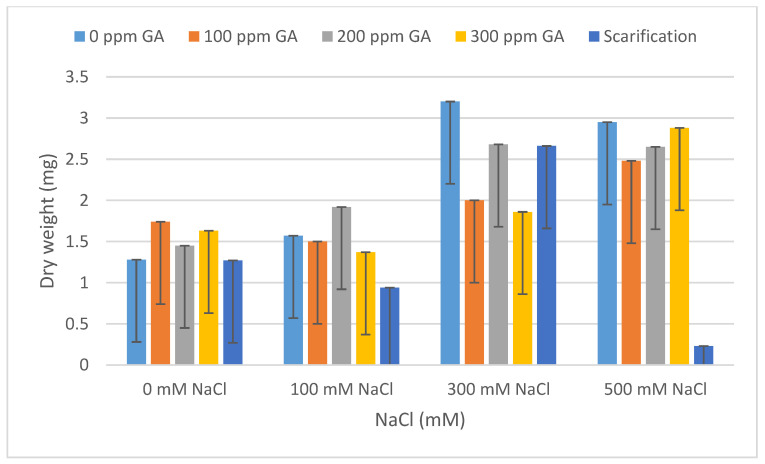
Effect of salinity, pre-treatment, and their interaction on dry weight (DW). Error bars indicate standard deviation. Means without * are not statistically significant differences according to Tukey’s test.

**Figure 7:**
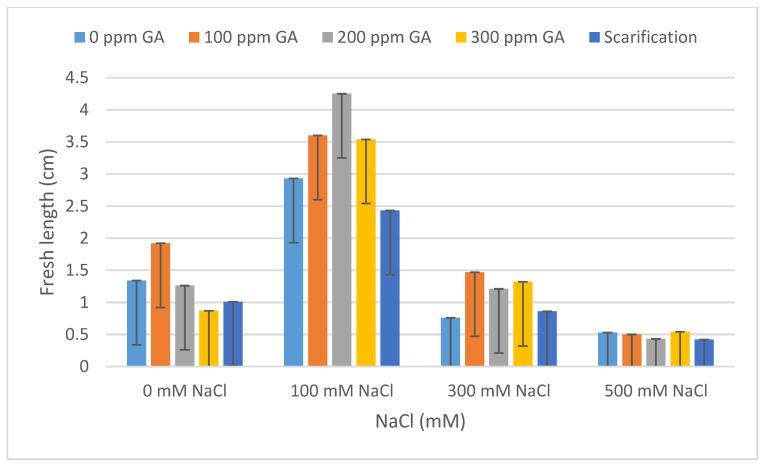
Effect of salinity, pre-treatment, and their interaction on fresh length (FL). Error bars indicate standard deviation. Means without * are not statistically significant differences according to Tukey’s test.

**Figure 8:**
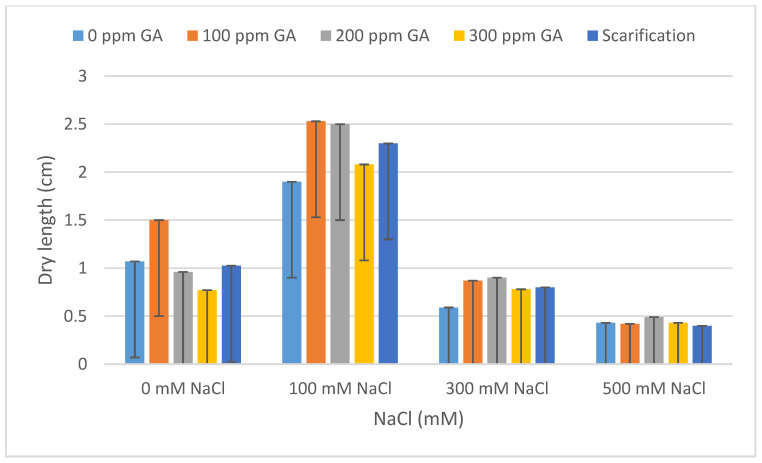
Effect of salinity, pre-treatment, and their interaction on dry length (DL). Error bars indicate standard deviation. Means without * are not statistically significant differences according to Tukey’s test.

**Figure 9:**
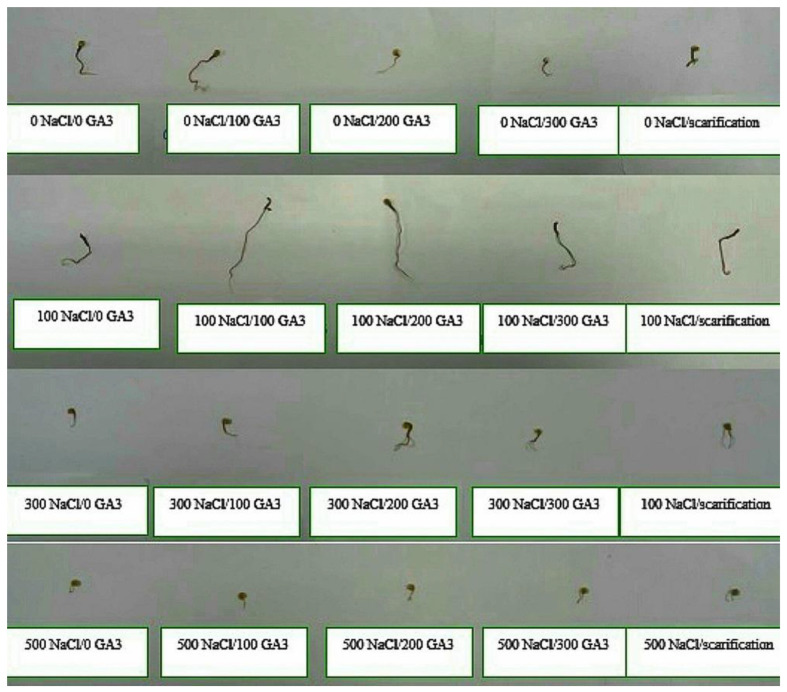
Effect of salinity, pretreatments, and their interaction on the germination of dried C. quinoa seedlings.

According to Table 4 and Table 5, the lowest fresh and dry weight and fresh and dry length values of *C. quinoa* seeds after 5 days of germination were observed with mechanical scarification under 500 mM NaCl (2.62 mg, 0.45 mg, 0.92 cm, and 0.67 cm, respectively). Furthermore, the highest fresh weight and fresh length values were obtained at 200 ppm of GA3 under 100 mM NaCl, at 300 mM of NaCl without pretreatment for dry weight, and at 100 ppm of GA3 under 100 mM of NaCl for dry length (10.96 mg, 1.92 mg, 2.91 cm, and 1.46 cm, respectively) (Figure 5, Figure 6, Figure 7, Figure 8 and Figure 9).

## 3. Discussion

### 3.1. Effect of GA3

GA3 promoted seed germination by modulating the synthesis of the enzyme alpha-amylase, which participates in the hydrolysis of starch, converting it into simple sugars. Subsequently, these sugars are translocated to locations where they are needed for growth [22]. In addition, ref. [23] suggests that GA3 improves the plasticity of the cell wall and increases water uptake.

In contrast, GA3 alone did not result in a high germination rate. This may be attributed to variations in the genotype and ecological conditions of seed production [24]. It is also possible that the concentrations of GA3 used in this study are not optimal for influencing the germination parameters studied. Similarly, ref. [25] found that the alleviation of the primary seedling dormancy of *Salicornia rubra* (Chenopodiaceae) seeds was not affected by GA3.

### 3.2. Effect of Salinity

The data obtained in the present study concerning salinity may be a result of increased NaCl stress during the germination process [26]. Salt stress induced a delay or the prevention of seed germination via several factors: For example, the synthesis of ethylene is inhibited during imbibition [27]. Therefore, the stored reserves are subject to change, and the availability of water can be reduced (osmotic stress) [28,29]. In addition, the activities of certain enzymes or hormones in the seed may be altered by an excess of Na^+^ ions [26,30]. The toxic effect of Na^+^, together with Cl^−^, could cause a reduction in cell elongation [31] and prevent the development of radicles through the seed coat as the shoot grows [32].

Nevertheless, one of the mechanisms used by *C. quinoa* to tolerate salt stress and maintain ionic hemostasis during germination and seedling establishment is to accumulate sodium and maintain potassium in the cytosol [33]. Moreover, the epidermal bladder cells and stomatal patterning of *C. quinoa* are acknowledged as notable contributors to salt stress tolerance [34]. Salt stress exerts detrimental effects on plants across various growth stages, thus hindering seed germination, growth, development, flowering, and fruiting [35]. Typically, stress resistance improves as plants mature. Nevertheless, even in halophytes, the impact of salt stress on plant development is often most severe during the early stages when plant sensitivity is at its peak [36,37,38]. Otherwise, during flowering, *C. quinoa* plants adapt to salt stress by adjusting the Na^+^ concentration of the root to that of the root medium and reducing Cl^−^ levels, which facilitates water uptake [39].

Studies have reported that Quinoa exhibits a combination of physiological attributes that confer remarkable salt tolerance [40,41]. Concentrations ranging from 150 to 250 mM NaCl have been shown to delay the onset of seed germination in *C. quinoa* [42]. Nevertheless, exposure to salt stress at concentrations of 100 mM NaCl and 200 mM NaCl did not result in negative effects on seed germination and growth, which corroborates our observations. However, at a concentration of 300 mM NaCl, substantial perturbations in the water balance of plants ensued due to cellular structural damage [43].

Our findings parallel those of [44], which demonstrated that quinoa exhibits tolerance to medium (150 mM) up to elevated (750 mM NaCl) concentrations of NaCl. Similarly, ref. [45] reported comparable outcomes, indicating that shoot lengths decreased by 60% at 300 mM NaCl in three out of four quinoa genotypes studied. According to [46] among the cultivars studied, the Titicaca variety exhibited the highest sensitivity to salt stress, manifesting the lowest germination rate. The authors of [16] hypothesized that environmental factors may influence genes associated with the germination process.

### 3.3. Effect of Interaction between GA3 and Salinity

The results show an interesting finding where the combined application of GA3 with different salinity levels improves the germination characteristics of quinoa seeds.

According to [47], the reduction in salt stress by GA3 could be attributed to the activation of enzymes acting in RNA and protein synthesis. In addition, GA3 potentially modulates stress responses by promoting sucrose transportation from the cotyledons to the shoots and by signaling an increase in invertase activity in the shoots [48].

Reciprocities in findings were noted in [23], which described that GA3 under 300 mM NaCl elevated the germination percentage and mitigated the adverse impact of salt stress on germination. Also, ref. [49] showed that even at high salinity (100 mM), seeds treated with 150 ppm of GA3 showed an increase in shoot length and weight with respect to oat cultivars. Likewise, the external application of GA3 is acknowledged in its mitigation of the impacts of salinity, and it facilitates germination in certain halophytic seeds, such as *Saticornia rubra* (Chenopodiaceae), *Crithmum maritimum* (Apiaceae), *Allenrolfea occidentalis* (Chenopodiaceae), *Arthrocnemum indicum* L., and *Chenopodium quinoa* [25,50,51,52].

However, despite this observation, it is important to note that the effect of the interaction between salinity and GA3 can be influenced by several factors, such as the concentrations of salinity and GA3, cultivars, grain development stage, etc.

### 3.4. Effect of Interaction between MS and Salinity

The seed coat can impede seed germination by physically restricting embryo growth. Seed scarification is a pathway employed to rupture the seed coat, thereby improving seed germination and subsequent plant development under challenging environmental conditions [53]. Ref. [54] also reported that scarification can break dormancy and increase the germination of seeds.

However, ref. [55] stated that severe mechanical scarification with sandpaper can cause damage to the embryo, which may result in some unviable embryos. Furthermore, ref. [56] reported that salinity can damage sacrificed embryos, and it has an effect on seedling growth. Salinity stress can also lead to secondary seed dormancy. In fact, ref. [56] also observed that for two species of Chenopodiaceae, *Suaeda physophora* and *Haloxylon ammodendron,* scarification did not affect seed water uptake and total germination.

Given these results, it is important to avoid the mechanical scarification of quinoa seeds as a pretreatment method. Alternative pretreatment methods should be investigated to improve germination parameters and ensure normal plant development, especially under saline conditions.

## 4. Materials and Methods

### 4.1. Plant Material

The certified seeds of *Chenopodium quinoa* (Titicaca variety) utilized in this study were harvested in August 2023 from northeastern Morocco, Berkane province. Seeds were provided by the University of Copenhagen, Denmark [57], within the BAFI/BYU-IAV Hassan II project. For this experiment, seeds were sown in Petri dishes.

### 4.2. Treatments

The experiment was designed to evaluate the effects of salinity (NaCl) and gibberellic acid (GA3), mechanical scarification (MS), and a combination of pretreatments NaCl/GA3 and NaCl/MS on the germination parameters of *C. quinoa* seed. Each treatment represents a specific combination of the modalities of the variables studied (Table 6).

The seeds selected were sterilized for 3 min with 12% sodium hypochlorite diluted to 10%; subsequently, the seeds were rinsed with distilled water to eliminate any residual traces of sodium hypochlorite.

Two pretreatments were used: The first was soaking seeds in different concentrations of gibberellic acid (GA3) (100, 200, and 300 ppm) at 20 °C for 2 h, and the second was mechanical scarification. The seeds were placed on sandpaper and rubbed along the abrasive surface with gentle but firm pressure.

Following this, the seeds were directly placed into Petri dishes measuring 10 cm in diameter, which contained filter paper disks that had been sterilized and saturated with distilled water to uphold optimal moisture levels. For salt stress, treatments using 5 mL of NaCl for each concentration prepared (0: distilled water; 100, 300, and 500 mM) were added to the Petri dishes and subsequently positioned within a controlled environmental growth chamber maintained in darkness at a temperature of 25 °C. The light cycle consisted of 16 h of illumination followed by 8 h of darkness, with a relative humidity of 70%. The experiment lasted for a duration of 5 days.

### 4.3. Seed Germination and Design of Experiments

The experimental design adopted for this experiment is a complete randomized design with three replications. Fifty seeds were used for each Petri dish and each repetition. The duration of the germination process was 5 days. We counted the germination rate daily. Seeds were considered germinated when the length of the radicle surpassed 2 mm. The germination process was initiated with the emergence of the primary radicle and succeeded by the development of the hypocotyl and root hairs, ultimately leading to cotyledon emergence [58].

### 4.4. Measured Parameters

Germination characteristics evaluated were as follows:

1. Total germination (TG) is measured according to the following formula [59].

TG (%) = (Total number of seeds germinated on day 5/number of total seeds) × 100,

2. Vigor index (VI) is calculated according to the following formula [60].

VI = (mean seedling length (cm) × the final germination percentage on day 5)/100,

3. Mean germination time (MGT) is measured using the following formula [61].

MGT = ∑ (Seeds germinated on each day × Number of days since the start of the experiment)/Total seeds germinated during the experiment),

4. Germination index (GI): The formula from [62] is used to measure the germination index (GI).

GI = Σ (Number of seeds germinated per day/Day of observation),

5. The length and weight of fresh and dry germinated seeds were determined as follows.
  Five days after germination, the length of the seedlings was measured using a millimeter ruler.

A precision analytical balance, with an exactitude of 0.001 g, was utilized to ascertain the fresh and dry masses of the seedlings. The dry mass was obtained by subjecting the material to desiccation in a forced-air oven maintained at 65 ± 5 °C for a duration of 48 h, as per [63].

### 4.5. Statistical Analysis

The data underwent statistical analysis using a two-way ANOVA following the arcsine transformation of all percentage values. NaCl levels and pre-treatments were considered as factors. The data generated were analyzed. Means were compared using the Tukey test. A post hoc test was used to determine significant differences between multiple groups, and the values obtained for each interaction were compared with the control.

The statistically significant difference was established using specific *p*-value levels: *p* ≤ 0.05 was considered statistically significant, *p* ≤ 0.01 was considered highly significant, and *p* ≤ 0.001 was considered very highly significant. According to these levels, the symbols *, **, and *** have been used in the figures and tables to indicate the level of statistically significant differences when *p* ≤ 0.05, *p* ≤ 0.01, and *p* ≤ 0.001, respectively. The graphs were drawn using Excel software for Microsoft 365 MSO, Version 2405 Build 16.0.17628.20006, and all the results were compared with the control.

## 5. Conclusions

In this study, it was observed that applying gibberellic acid improves germination parameters even under high salt concentrations (300 mM of NaCl).

On the other hand, germination parameters were significantly decreased by mechanical scarification under high concentrations of NaCl (500 mM).

These results reveal that the Titicaca cultivar showed its best performance at 100 mM NaCl. This suggests that Titicaca shows a remarkable ability to adapt to saline environments.

Furthermore, this work demonstrates that the application of gibberellic acid has the potential to improve the germination parameters of C. *quinoa* seeds under salinity stress. However, the application of these methods poses several challenges, such as the cost of application at the industrial level, the technical expertise required to apply these practices, and the genetic variability of seeds produced each year, which makes it difficult to determine the optimal application method.

These results are very promising for selecting cultivars adapted to difficult environmental conditions. Finally, avenues for future research must be carried out to study the physiological, cellular, molecular, and physiological behavior of quinoa with the different pre-treatment under salinity stress.

## Figures and Tables

**Table 1:** Analysis of variance for the effect of pretreatments, salinity, and their interaction on quinoa germination parameters.

Source of Variation	Df ^1^	TG ^2^	VI ^3^	FW ^4^	DW ^5^	FL ^6^	DL ^7^	MGT ^8^	GI ^9^
Pretreatments (gibberellic acid+ scarification)	4	ns ^13^	**	ns	ns	ns	ns	ns	ns
Salinity	3	***	***	*** ^12^	ns	** ^11^	* ^10^	ns	***
Pretreatments × salinity	12	ns	ns	ns	ns	ns	ns	ns	ns

^1^: Degree of freedom; ^2^: total of germination; ^3^: vigor index; ^4^: fresh weight; ^5^: dry weight; ^6^: fresh length; ^7^: dry length; ^8^: mean germination time; ^9^: germination index; ^10^: Significant at *p* ≤ 0.05; ^11^: highly significant at *p* ≤ 0.01; ^12^: very highly significant at *p* ≤ 0.001; ^13^: not significant.

**Table 2:** Effect of salinity, pre-treatments, and their interaction on total germination and the vigor index compared to the control. Means are presented as ± standard deviation. Statistically significant differences are indicated by *, **, and *** when *p* ≤ 0.05, *p* ≤ 0.01, and *p* ≤ 0.001, respectively. Means without * are not statistically significant differences according to Tukey’s test.

Pretreatment	Salinity	Mean TG (%)	Increase	Decrease	Mean VI	Increase	Decrease
0 GA	0 NaCl (control)	84.00 ± 8.66	-		1.13 ± 0.56	-	-
	100 NaCl	75.33 ± 3.51	-	8.57	2.21 ± 0.32 **	1.08	-
	300 NaCl	53.33 ± 6.02 *	-	30.57	0.41 ± 0.07 *	-	0.72
	500 NaCl	21.33 ± 5.13 ***	-	62.67	0.11 ± 0.04 **	-	1.02
100 GA	0 NaCl	89.33 ± 11.01	5.33	-	1.72 ± 0.24	0.59	-
	100 NaCl	75.33 ± 13.45	-	8.67	2.71 ± 0.67 ***	1.58	-
	300 NaCl	81.33 ± 7.63	-	2.67	1.20 ± 0.08	0.07	-
	500 NaCl	20.67 ± 5.13 ***	-	63.33	0.10 ± 0.04 **	-	1.03
200 GA	0 NaCl	79.67 ± 5.13	-	4.33	0.99 ± 0.19	-	0.14
	100 NaCl	76.00 ± 7.50	-	8.00	3.23 ± 0.10 ***	2.10	-
	300 NaCl	84.00 ± 6.24	-	-	1.02 ± 0.08	-	0.11
	500 NaCl	22.00 ± 9.50 ***	-	62.00	0.09 ± 0.04 **	-	1.04
300 GA	0 NaCl	74.67 ± 5.13	-	9.33	0.65 ± 0.02	-	0.48
	100 NaCl	78.00 ± 5.00	-	6.00	2.76 ± 0.13 ***	1.63	-
	300 NaCl	82.00 ± 8.74	-	2.00	1.08 ± 0.10	-	0.05
	500 NaCl	39.33 ± 12.12 **	-	44.67	0.21 ± 0.06 **	-	0.92
Scarification	0 NaCl	78.67 ± 6.93	-	5.33	0.79 ± 0.57	-	0.34
	100 NaCl	70.67 ± 5.57	-	13.33	1.72 ± 0.34	0.59	-
	300 NaCl	41.33 ± 8.54 **	-	42.67	0.36 ± 0.09 *	-	0.77
	500 NaCl	15.33 ± 3.60 ***	-	68.67	0.06 ± 0.03 **	-	1.07

**Table 3:** Effect of salinity, pre-treatments, and their interaction on the mean germination time and germination index compared to the control. Means are presented as ± standard deviation. Statistically significant differences are indicated by ** when *p* ≤ 0.01. Means without * are not statistically significant differences according to Tukey’s test.

Pretreatment	Salinity	Mean MGT (Day)	Increase	Decrease	Mean GI	Increase	Decrease
0 GA	0 NaCl (control)	3.26 ± 0.03	-	-	141.08 ± 7.07	-	-
	100 NaCl	3.17 ± 0.01	-	0.09	142.40 ± 1.41	1.32	-
	300 NaCl	3.32 ± 0.06	0.06	-	82.44 ± 2.83	-	58.64
	500 NaCl	3.64 ± 0.03	0.38	-	23.21 ± 4.24 **	-	117.87
100 GA	0 NaCl	3.21 ± 0.01	-	0.05	152.42 ± 5.66	11.34	-
	100 NaCl	3.17 ± 0.01	-	0.09	141.96 ± 2.83	-	0.88
	300 NaCl	3.32 ± 0.03	0.06	-	128.04 ± 4.24	-	13.04
	500 NaCl	3.70 ± 0.03	0.44	-	21.13 ± 2.83 **	-	119.95
200 GA	0 NaCl	3.09 ± 0.03	-	0.17	161.34 ± 8.49	20.26	-
	100 NaCl	3.20 ± 0.01	-	0.06	138.09 ± 4.24	-	2.99
	300 NaCl	3.26 ± 0.03	-	-	142.74 ± 2.83	1.66	-
	500 NaCl	3.65 ± 0.03	0.39	-	24.57 ± 4.24 **	-	116.51
300 GA	0 NaCl	3.11 ± 0.06	-	0.15	149.71 ± 5.66	8.63	-
	100 NaCl	3.15 ± 0.03	-	0.11	145.42 ± 4.24	4.34	-
	300 NaCl	3.30 ± 0.01	0.04	-	133.57 ± 4.24	-	7.51
	500 NaCl	3.70 ± 0.03	0.44	-	39.81 ± 1.41 **	-	101.27
Scarification	0 NaCl	3.11 ± 0.01	-	0.15	157.73 ± 4.24	16.65	-
	100 NaCl	3.48 ± 0.03	0.22	-	92.30 ± 1.41	-	48.78
	300 NaCl	3.32 ± 0.01	0.06	-	65.82 ± 2.83	-	75.26
	500 NaCl	3.49 ± 0.03	0.23	-	19.79 ± 1.4 **	-	121.29

**Table 4:** Effect of salinity, pre-treatment, and their interaction on fresh weight and dry weight compared to the control. Means are presented as ± standard deviation. Statistically significant differences are indicated by ** when *p* ≤ 0.01. Means without * are not statistically significant differences according to Tukey’s test.

Pretreatment	Salinity	Mean FW (mg)	Increase	Decrease	Mean DW (mg)	Increase	Decrease
0 GA	0 NaCl (control)	3.78 ± 0.21	-	-	1.28 ± 0.01	-	-
	100 NaCl	9.01 ± 0.42	5.23	-	1.57 ± 0.16	0.29	-
	300 NaCl	5.00 ± 0.67	1.22	-	3.20 ± 0.12	1.92	-
	500 NaCl	6.14 ± 0.28	2.36	-	2.95 ± 0.01	1.67	-
100 GA	0 NaCl	4.77 ± 0.17	0.99	-	1.74 ± 0.37	0.46	-
	100 NaCl	8.47 ± 0.49	4.69	-	1.50 ± 0.36	0.22	-
	300 NaCl	7.82 ± 0.14	4.04	-	2.00 ± 0.45	0.72	-
	500 NaCl	5.37 ± 0.59	1.59	-	2.48 ± 0.07	1.20	-
200 GA	0 NaCl	2.28 ± 0.29	-	1.50	1.45 ± 0.04	0.17	-
	100 NaCl	14.74 ± 0.47 **	10.96	-	1.92 ± 0.04	0.64	-
	300 NaCl	7.46 ± 0.48	3.68	-	2.68 ± 0.28	1.40	-
	500 NaCl	4.63 ± 0.13	0.85	-	2.65 ± 0.18	1.37	-
300 GA	0 NaCl	2.52 ± 0.79	-	1.26	1.63 ± 0.09	0.35	-
	100 NaCl	9.12 ± 0.12	5.34	-	1.37 ± 0.14	0.09	-
	300 NaCl	7.43 ± 0.51	3.65	-	1.86 ± 0.18	0.58	-
	500 NaCl	6.75 ± 0.39	2.97	-	2.88 ± 0.12	1.60	-
Scarification	0 NaCl	2.48 ± 0.48	-	1.30	1.27 ± 0.04	-	0.01
	100 NaCl	4.65 ± 0.34	0.87	-	0.94 ± 0.14	-	0.34
	300 NaCl	7.27 ± 0.28	3.49	-	2.66 ± 0.30	1.38	-
	500 NaCl	1.16 ± 0.29	-	2.62	0.23 ± 0.10	-	1.05

**Table 5:** Effect of salinity, pre-treatment, and their interactions on fresh length and dry length compared to the control. Means are presented as ± standard deviation. Means without * are not statistically significant differences according to Tukey’s test.

Pretreatment	Salinity	Mean FL (cm)	Increase	Decrease	Mean DL (cm)	Increase	Decrease
0 GA	0 NaCl (control)	1.34 ± 0.56	-	-	1.07 ± 0.13	-	-
	100 NaCl	2.93 ± 0.40	1.59	-	1.90 ± 0.02	0.83	-
	300 NaCl	0.76 ± 0.04	-	0.58	0.59 ± 0.09	-	0.48
	500 NaCl	0.53 ± 0.06	-	0.81	0.43 ± 0.04	-	0.64
100 GA	0 NaCl	1.92 ± 0.04	0.58	-	1.51 ± 0.13	0.44	-
	100 NaCl	3.60 ± 0.20	2.26	-	2.53 ± 0.05	1.46	-
	300 NaCl	1.47 ± 0.04	0.13	-	0.87 ± 0.09	-	0.20
	500 NaCl	0.50 ± 0.10	-	0.84	0.42 ± 0.05	-	0.65
200 GA	0 NaCl	1.27 ± 0.24	-	0.07	0.96 ± 0.1	-	0.11
	100 NaCl	4.25 ± 0.26	2.91	-	2.50 ± 0.13	1.43	-
	300 NaCl	1.21 ± 0.15	-	0.13	0.90 ± 0.04	-	0.17
	500 NaCl	0.43 ± 0.08	-	0.91	0.49 ± 0.08	-	0.58
300 GA	0 NaCl	0.86 ± 0.05	-	0.48	0.77 ± 0.10	-	0.30
	100 NaCl	3.54 ± 0.10	2.20	-	2.08 ± 0.07	1.01	-
	300 NaCl	1.32 ± 0.02	-	0.02	0.78 ± 0.03	-	0.29
	500 NaCl	0.54 ± 0.04	-	0.80	0.43 ± 0.05	-	0.64
Scarification	0 NaCl	1.01 ± 0.55	-	0.33	1.02 ± 0.08	-	0.05
	100 NaCl	2.44 ± 0.26	1.10	-	2.30 ± 0.03	1.23	-
	300 NaCl	0.86 ± 0.12	-	0.48	0.76 ± 0.02	-	0.31
	500 NaCl	0.42 ± 0.04	-	0.92	0.40 ± 0.06	-	0.67

**Table 6:** Variables, modalities, and their treatments used in the experiment.

	Variables		Treatment
	Pretreatments: GA3 (ppm) and MS	Salinity (NaCl) mM	
Modalities	0 GA3 100 GA3 200 GA3 300 GA3 MS	0	0/0 100/0 200/0 300/0 MS/0
	0 GA3 100 GA3 200 GA3 300 GA3 MS	100	0/100 100/100 200/100 300/100 MS/100
	0 GA3 100 GA3 200 GA3 300 GA3 MS	300	0/300 100/300 200/300 300/300 MS/300
	0 GA3 100 GA3 200 GA3 300 GA3 MS	500	0/500 100/500 200/500 300/500 MS/500

## Data Availability

Data are contained within the article.
